# What would reduce caesarean section rates?—Views from pregnant women and clinicians in Ireland

**DOI:** 10.1371/journal.pone.0267465

**Published:** 2022-04-28

**Authors:** Louise Gallagher, Valerie Smith, Margaret Carroll, Kathleen Hannon, Denise Lawler, Cecily Begley

**Affiliations:** 1 School of Nursing & Midwifery, Trinity College Dublin, Dublin, Ireland; 2 Health Information and Quality Authority, Dublin, Ireland; Ordu University, TURKEY

## Abstract

**Background:**

Caesarean section rates continue to rise in most parts of the world. While CS is a lifesaving procedure there is evidence that, beyond a certain threshold, CS rates may contribute to increased maternal and perinatal morbidity. This study aimed to elicit the views of pregnant women’s and clinicians’ on how CS rates might be reduced.

**Methods:**

Pregnant women and their partners, and clinicians working with pregnant women in a maternity hospital in the Republic of Ireland of Ireland, were invited to participate in focus groups. Eligibility criteria included all women attending antenatal classes and clinicians working with pregnant women. A convenience sample was used and interviews were audio recorded, transcribed, and analysed using thematic analysis.

**Results:**

Four focus group interviews were conducted with 30 clinicians and 15 pregnant women and two partners participated in three focus groups. A further two women were interviewed individually. Participants expressed a view that rising CS rates were impacted by a societal perception that CS had become a ‘normal mode of birth’. Suggestions for reducing CS rates were offered by clinicians and pregnant women and their partners.

**Conclusions:**

Clinicians and pregnant women consider that CS rates can be reduced if a shared philosophy supporting normal birth is prioritised alongside adequate resourcing. Women and their partners also believe that enhanced communication with clinicians is central to reducing CS rates.

## Introduction

Caesarean section (CS) rates are rising across the world, causing widespread concern [1]. In Europe, rates vary from 16% in Iceland to 59% in Cyprus [2]. The CS rate in Ireland, based on 2018 data, is 31% and similar rates exist in many other high-income countries including; Australia (30%), United States (33%), and Brazil (55%) [2]. These high rates occur in the context of evidence from the World Health Organization (WHO) that CS rates above 10–16% in any population do not result in a decrease in mortality rates for women or infants, which indicates that some CSs in those countries may be unnecessary [3, 4].

Caesarean section is a major surgical procedure with higher rates of maternal mortality and maternal morbidity than after vaginal birth. For the mother, CS is associated with an increased risk of ectopic pregnancy, preterm birth, stillbirth, uterine rupture, and abnormal placentation [5]. Short-term risks of CS for infants include an increased likelihood of allergic disorders, atopy, and asthma, altered immune development, and reduced diversity of intestinal gut microbiome [5]. These associated risks and the costs associated with the procedure have led to calls from the WHO and the International Federation of Gynaecology and Obstetrics (FIGO) to reduce rates of unnecessary CS [3].

Recently, FIGO published a position paper on CS rates, expressing their concern by saying: *“Worldwide there is an alarming increase in caesarean section rates*.*”* They stated that *“The large variation in CS rates indicates that these rates have virtually nothing to do with evidence-based medicine*,*”* and ended by appealing for the help of *“governmental bodies*, *UN partners*, *professional organisations*, *women’s groups*, *and other stakeholders to reduce unnecessary CSs*.*”* [6].

As part of an ongoing research initiative aimed at developing an evidence-based intervention for reducing unnecessary caesareans safely, we conducted focus group interviews to ascertain pregnant women’s and clinicians’ views on caesarean sections and how they felt the rate of CS could be reduced, while also discussing vaginal births following previous CS (VBACs) and how they might be encouraged.

## Methods

### Aim

To ascertain pregnant women’s and clinicians’ views on caesarean sections and their views on how CS rates might be reduced.

### Setting

This study was conducted in the Republic of Ireland, in a single maternity hospital which has over 8,000 births annually and had a CS rate of 31% in 2017. The latest annual Clinical Report shows that the CS rate in 2019 is 33.8%, with a VBAC rate varying between 19.3% for women with one previous CS and 46.8% for women with one previous CS and at least one vaginal birth.

### Participants and participant recruitment

Following ethical approval from both the Research Ethics Committee of the Faculty of Health Sciences, Trinity College Dublin and the study site, an exploratory qualitative design was used. Consenting women, their partners and clinicians were recruited by gatekeepers (midwives working in antenatal education). All pregnant women attending antenatal classes and all clinicians working with pregnant women at the study site were eligible to participate. Information about the study was distributed by gatekeepers to clinicians in clinical areas and to pregnant women at antenatal classes, at least one week prior to the interviews. Convenience sampling was used in that all volunteers who could attend the focus group interviews (FGIs) at the scheduled time were invited to join. Written informed consent was taken at the time of interview.

### Data collection and analysis

Four focus group interviews were conducted with 30 clinicians comprising two groups of six persons, one with five and one with thirteen. Participants included eighteen midwives, nine obstetricians, and two physiotherapists (one missing data). Twelve of the clinicians reported working in their current professional field for more than 10 years, while seven had >5 to 10 years and an additional seven had worked for 2–5 years. One clinician had less than 2 years’ experience (n = 27). Fifteen pregnant women (11 nulliparous, four multiparous) and two partners were interviewed, 15 of them in three focus groups (four, five and six participants), and two women in individual interviews as they expressed an eagerness to participate but could not attend the scheduled focus groups. Two women were under the age of 30, five women and one partner were aged 31–35, and seven women and one partner were between 36 to 40 years of age.

The interview schedule was semi-structured, consisting of 8 open-ended questions (Box 1).

**Box 1**

**Interview schedule for clinicians’ interviews**

Do you think the CS rate in this hospital is at too low a rate, about right, or too high? And why?

What factors are important if we would like to reduce CS rates?

And what factors are important to encourage more VBACs?

What are the barriers to vaginal birth and VBAC?

What is important to you as a professional?

What are your views on shared decision-making with women?

How can women be supported to be confident to have a vaginal birth?

Is there anything else you would like to add?

**Interview schedule for women/partner’s interviews**

Do you think this hospital’s rate is too low, about right, or too high? And why?

What factors are important to you to try not to have a CS?

And what factors are important to you if you had a CS before, to try to help you to have a VBAC?

What are the barriers that prevent you from having a vaginal birth or a VBAC?

What is important to you as a woman, or as a woman’s partner?

What do you think about having shared decision-making with clinicians?

What would you like done to support you to have a vaginal birth?

Is there anything else you would like to add?

Clinician interviews took place in an education facility adjacent to the hospital and women were interviewed in a center where antenatal education is provided. Interviews with clinicians ranged from 33 minutes to 57 minutes, and interviews with women ranged from 28 minutes (individual interview) to 49 minutes (group interview). All interviews were audio recorded, transcribed, and analysed separately by two researchers, using thematic analysis [7]. A copy of transcripts was made available to participants on request. Analysis involved three researchers reading the transcripts independently to identify key categories and recurrent themes concerning views on caesarean sections and how CS rates might be reduced. Sections of text were marked and linked to sections of text from other interviews that covered similar issues or experiences. Emerging themes were jointly reviewed and interpretations were subsequently discussed and challenged until agreement was reached. Data analysis continued until no new themes or ideas were emerging.

## Findings

Thematic analysis resulted in four main themes; ‘Caesarean section has become normalised’ was the central core, shared theme consistent between clinicians and pregnant women, and it contained two subthemes (Table 1). The other three themes were ‘Factors increasing the CS rate’, ‘Suggestions for reducing the CS rate’, and ‘Enhanced communication and relationship with clinicians is needed’.

**Table 1:** Themes and sub-themes.

Women’s/partners’ and clinicians’ shared views
**Theme 1:** **Caesarean section has become normalised** **Sub-themes:** a. Social and mainstream media influence b.Better antenatal preparation and care required **Theme 2:** **Factors increasing the CS rate** **Theme 3:** **Suggestions for reducing CS rates** **Sub-themes:** a.Changing the culture b.Need for resources and support c.Need for a shared philosophy d.Practical ways to reduce CS rates **Theme 4: Enhanced communication and relationship with clinicians is needed**

### 1. Caesarean section has become normalised

This was the central, core theme, expressed by women, their partners and clinicians. All participant groups believed that rising CS rates were being influenced by a societal perception that CS had become a ‘normal mode of birth’. Clinicians expressed concern that increasing rates led to women underestimating the risks associated with the procedure, and women spoke about the perception that vaginal birth was more risky:

*Because it’s normal, they have them all the time. But I think they fail to realise, like it’s major abdominal surgery that you’re having…that’s why people are just so blasé about it. It’s like ‘ah, sure, I’ll just have a section.’ (Clinician FGI 1)*

*I know friends that have a…problem with bladder control and they say therefore I want a C section because when I give vaginal birth, then I will have problem with my bladder afterwards (Woman FGI 1)*.

This perception was echoed by pregnant women and their partners:

*So those percentages [CS rates] just seem kind of normal to me. (Woman FGI 3)*

Women noted that this was a shift from the experiences of the previous generation:

*I think that’s nearly one in three is going with a section…I think my mam and my aunties, do you know that [CS] just never happened for them. (Woman FGI 3)*

**a. Social and mainstream media influence.** Central to the participants’ view that CS was being normalised was increasing portrayals on popular and social media of women giving birth by CS. Emphasised in such stories was a focus on the birth and they felt there was a lack of balance around the potential risks or consequences:

*In the media and, you know, celebrities and that, seeing all these people having elective sections and… they don’t hear about the risks or the people losing their uterus or people bleeding… (Clinician FGI 4)*

Representations of CS were believed by women and partners to be minimising the impact on women:

*…on TV and movies… the woman, she looks like she’s awake, she’s fine. She doesn’t feel any pain. She can chat. And maybe that affects it. (Woman Interview 1)*

*People don’t understand…. like [name of pregnant partner] could get condescending remarks sometimes… “but sure you had a section, it was fine!” They have no idea, none whatsoever at all. (Partner FGI 3)*

Women and clinicians shared the view that mainstream media and social media contributed to fears around normal birth, leading to women requesting or considering a caesarean birth:

*I’m… sort of worried about the whole process [of birth]…so I suppose I’d be sort of more, maybe more open to medical intervention because I have that little bit of fear that maybe other complications might [occur]… that’s why I would sort of look at it as a safe option, if it came to it. (Woman FGI 2)*

Clinicians also feared litigation arising from medical negligence cases, with its concomitant adverse media attention, and acknowledge that this is contributing to a rise in CS rates and a loss of confidence in normal birth:

*They’re [CSs] also generated from fear …..they’ve [pregnant women] lost confidence in the medical profession or the midwifery profession and I do think that we as practitioners and midwives have lost confidence too, I think we are more afraid than we ever have been… so we feel like we’re under surveillance all the time and we are being criticised by ourselves, by our colleagues, by our media, by women. (Clinician FGI 2)*

**b. Better antenatal preparation and care required.** Clinicians and women identified enhanced antenatal education as an opportunity to oppose the normalising of CS, promote confidence in normal birth and counter women’s anxieties:

*A lot of women don’t go to antenatal classes here … If the women are educated, if they know bits and pieces… they’re less afraid. (Clinician FGI 1)*

This was also echoed by women who wanted early education to process and facilitate decision making:

*I think you actually need information, more information at the beginning of the pregnancy…so that you do have plenty of time….if you can have the information given sooner. So you have time to process it. And really decide what’s best for you. Then I think that may help. (Woman FGI 3)*

Women who had attended classes noted increased confidence in normal birth, but also wanted enhanced access to other strategies such as hypnobirthing classes, which were not routinely available to them:

*[Hypnobirthing classes] should be as regular as these antenatal classes…at the start I was terrified of it [birth], but it’s only now that I’m getting a bit more information…showing it for the natural process that it is, you know… (Woman FGI 2)*

Women identified a need to have better preparation to increase confidence in their ability to make decisions around birth and communicate more openly with their caregivers. Nulliparous women, in particular, described feeling rushed through a ‘*conveyor belt’* system when attending antenatal appointments, and not having the opportunity to discuss their pregnancy or labour:

*‘ [Study Site] is a very busy hospital…sometimes it feels like you’re in and out and you don’t have time to sit there and say to someone ‘I don’t know how I feel about this, I don’t understand what’s happening,’ you know…I don’t really feel that there’s an atmosphere there where I could stop…the consultant there for a few minutes to ask. (Woman FGI 2)*

### 2. Factors increasing the CS rate

The clinicians had clear views that induction of labour (1) was having an adverse impact on CS rates, when the process was seen to ‘fail’:

*Literally they are presenting for ‘cold’ inductions…*[for vague indications] *at 38 weeks*, *39 weeks they are…not physiologically ready to labour and you are trying to force the process on them… that was one of the reasons for rising rates…that it’s failed inductions ending up in sections*. *(Clinician FG 4)*

*The induction rates are…going up all the time for reasons that are maybe, you know obstetrician going on holidays…one that really gets my goat here big time is gestational diabetes, even though there’s no macrosomic baby; so she’s got diabetes, like so what, she’s fine otherwise, you don’t have to induce her if the baby is not huge. (Clinician FG 1)*

Clinicians also considered that women presenting for IOL were ill-informed about the process involved and the possible outcomes. This incongruity between the process and women’s knowledge was perceived to contribute to pressure to perform a CS when pregnancy or labour was prolonged, and led clinicians to consider if they were failing women in their care:

*You can see them [women] thinking ‘this is too long’ when they are not informed…Have a look at the indications for CS, every one of them seems to say ‘failed induction’ or ‘failure to progress.’ Whose failure I wonder? Ours or theirs? (Clinician FG 3)*

Women also felt that having an IOL increased their chances of needing a CS, especially if labour was prolonged, and that many women desired this kind of intervention:

*I was slow to respond initially, obviously my waters were broken, it took me 7 hours to get to 3 centimetres and…I was aware, it was in the back of my mind ‘ok I haven’t reached my 3 centimetres yet.’ Now once I did things started to go quicker. But it still took 13 hours…you are aware of that, that you’re on the clock. (Woman FGI 1)*

*Medical intervention is high in priorities of other women, ‘get the pain killers, get the epidural’…(Woman FGI 2)*

Lack of experience or competence was also cited as a reason for increasing CS rates, which could be addressed by education of junior staff:

*Some of the obstetricians, the younger ones, they’re not learning how to do several things. I mean there’s very few of them can do a breech delivery anymore, so they have to do a caesarean…some of them can’t do a forceps delivery…they’re not learning the skills…their only option is to go to theatre. (Clinician FGI 1)*

*CTG analysis…is the big elephant in the room. (Clinician FGI 2)*

### 3. Suggestions for reducing CS rates

#### a. Changing the culture

In order to improve normal birth rates and halt rising CS rates, clinicians were clear that strong governance, leadership and accountability would be required to change the prevailing culture of normalised CS. While they felt that local initiatives could be successful for enhancing some practice initiatives, a more strategic approach would be required to reduce CS rates, that did not challenge senior obstetricians’ views:

*It’s just a matter of setting the tone and of all the senior people across all professions…the entire hospital saying firstly ‘is the section rate too high, yes or no?’ And making an agreed decision on that. And then if they feel ‘yes, it is’ well, how are we going to tackle it….it has to be driven by senior management within the hospital and nationally. (Clinician FGI 4)*

*No one…wants to say no to the consultants, you know, and there are very reasonable consultants who really are…quite strict on…who they book for induction, and then there are others that induce everybody…but the environment that we work in…we’re in a very, very small specialty…we as juniors move around the entire country all the time, but there’s very few of us, and nobody wants to upset anybody else. (Clinician FGI 3)*

Some clinicians recognised that they could participate more actively in women’s labours and support them in a more normal birth:

*C1 We should be saying if you want to optimise your physiology…you need to get up off the bed…this dependence on epidural analgesia is not always…the best for people…perhaps our industrialised model of maternity care is: ‘okay, woman comes in for induction, plug her in, CTG, epidural, Syntocin,’ we’re extremely comfortable with that…*

*C2 Get her quiet and asleep. (Clinician FGI 2)*

#### b. Need for resources and support

In order to redress the fear of litigation clinicians want a maternity care system that is fully resourced and designed to support birth properly:

*So it is I think a lot to do with resources, staffing, infrastructure, the system doesn’t currently support women to have a normal birth. (Clinician FGI 4)*

Adequate resources to support women’s choices, and provide both continuity and one to one support during birth were consistently identified as challenging in the current system of maternity care:

*Our staffing level has been very poor for the last ten…maybe twenty years and all the evidence would say, I mean it’s been proven again and again, that really a normal birth, it is …the midwife, that continuity of care, particularly in labour, and being with the woman…you can protect her and mind her… (Clinician FGI 3)*

That the midwife on the day is experienced and supportive of the fact that you do want to vaginally birth. And will encourage you to be in the correct positions for that and not be lying on your back and…having a midwife that’s going to be encouraging us to stay mobile. (Woman FGI 3)

#### C. Need for a shared philosophy

Central to the aim of increasing vaginal birth was the view from both clinicians and women, that a shared and common philosophy around normal birth can reduce rates of CS:

*It’s a philosophy, it’s an attitude and its fostered even from junior trainee midwives and doctors that if you come into a place where they are really concentrating on their section rates and really trying to let women labour themselves and labouring as normally and as intervention-free as possible it’s just a philosophy that you are brought up in. And that culture, as you move away from that, then it’s harder to pull it back. (Clinician FG4)*

Women tended to express the more negative view of this concept, emphasising a shared and common philosophy in society around the benefits of CS:

*But it’s people’s attitude as well, like there’s (laughs) you know like you get…“are you mad in the head that you want to try and have a vaginal birth. You can a lovely elective section.” (Woman FGI 3)*

Women also expressed that there was a difference in the philosophies and caring approaches of obstetricians and midwives:

*If you’re a private patient and your consultant…it’s late, and you’re taking forever; you know he’ll push you towards a C section. (Woman, individual interview 2)*

*W1: They [obstetricians] have a very different approach to the mothers I find, the midwives are far more relaxed, the way they speak to you…the consultants are terribly serious and it’s, it’s a very different experience*.

*W2: Midwives can be…they’re very sort of like, you know, ‘loads of women do this every day, it’s not a big deal,’…it sort of feels a bit more…natural, you know*.

*W3: Also they’re focused on the actual birth…that’s their area of expertise. The consultants…can be preoccupied with what can go wrong, do you know what I mean, rather than your actual birth. (Women FGI 2)*

Optimising the antenatal period to prepare women for birth was seen as pivotal to creating such a philosophy that supports normal birth and reduces caesarean section rates:

*I did the hypnobirthing class here on my previous pregnancy, I wish I’d been told about maybe starting it much earlier…And I really noticed when I was on antenatal wards before… other women…weren’t aware of information. Like basic things that they could have been told. (Woman FGI 1)*

#### d. Practical ways to reduce CS rates

The clinicians gave examples of a number of techniques they could use to encourage women to labour normally, and avoid a CS:

*C1 They maybe should be at home in early labour rather than being in the hospital…if you’re a woman who’s in pain and you don’t really know why you’re in pain and you think that you should be having your epidural and you’re told to go back to…room 5, you will complain*.

*C2 But even like what they were doing with the hopscotch, giving the women something to do in that time, I think that might actually really help…a distraction, you know. (Clinicians FG 2)*

Women also had some practical suggestions to reduce the CS rate such as staying at home as long as possible, keeping mobile, and remaining calm and relaxed, allowing the body’s natural hormones do their work:

*Your body is naturally producing hormones that only really work if you’re relaxed and if you’re in a certain state of mind where, you know, they can do their job, if you’re not then go into panic and maybe there’s an emergency C Section then, you know what I mean. (Woman FGI 2)*

### Theme 4: Enhanced communication and relationship with clinicians is needed

This was a core theme in the women’s and their partners’ FGIs, and clinicians also spoke about its importance and their need to improve. While women gave examples of shared decision-making with their caregivers in the antenatal period, they voiced concerns over their ability to share equal decision-making during their birth:

*I think…that you have to really fight for what you want when it comes to your birth…It doesn’t seem to me like [a pregnant woman is]…able to walk in and say ‘this is what I want’ and they go ‘okay that’s your birth plan, that’s what’s going to happen.’ I think she probably has to really fight for that. (Woman Interview 1)*

*Obviously…the midwives, or the obstetricians, know the best. You know and again because maybe I don’t trust myself enough. …I’m not going to insist and say, no I refuse (Woman Interview 2)*

While the importance of open communication between women and their healthcare providers was a theme that arose from FGIs with women, it did not arise as strongly from the clinicians’ FGIs. One clinician did touch upon a need for genuine engagement; however there was also some evidence of exerting professional dominance in order to influence women’s choices:

*It’s not trying to influence them unduly, it’s the fact that you are educated and trained in something and that you have seen loads of different outcomes that they haven’t seen. So I think…shared, informed decision making, for the woman… It’s not just giving someone the information and saying ‘do you understand that?’ and letting them come back, it’s genuinely engaging with them. (Clinician FGI 4)*

Women, however, clearly articulated a desire to have autonomy over their decisions including mode of birth:

*For me, I would feel like I want to have a level of control in any decision making. (Woman individual interview 1)*

Multiparous women spoke of a lack of clear communication in their previous pregnancy and birthing experience, or in their friends’ experiences, that negatively impacted on their ability to consent to medical intervention and on their involvement in the decision-making process:

*Like I had to have a sweep on my pregnancy and they just said they were going to do a medical exam normally…but if they had used that terminology to me I would have known…And it annoyed me afterwards, I didn’t mind, I said ‘yeah that’s ok’ but it was only afterwards I kind of said ‘I think that was a sweep.’ If I had been told… You are vulnerable, you’re vulnerable. (Woman FGI 1)*

*It wasn’t really fully explained to [my friend]…so there was this kind of steamrolling action where you lose all level of say, control, power, anything, in this situation. (Women FGI 2)*

*On my first pregnancy I was breech pretty much all along… And certainly then there was no facilitation of decision. It was just like ‘you’re having a section first thing tomorrow morning.’ And I just felt out of control like at that point…because then I’d gotten her to turn, I had like my hopes built up, like ‘okay, I’m going to have the birth that I wanted.’ Whereas then that was like taken away. (Woman FGI 3)*

Some clinicians were aware that communication by obstetricians in the antenatal period was not ideal, and led to increased IOL rates, which, they felt, could increase CS rates:

*C1 Yeah so we’d admit them to the ward…quite a large amount of the time, they don’t know the process, what they’re going to be subjected to or involved in and they… don’t really understand why they’re being induced*.

*C2 And they don’t understand the risks either, they’re not being told that it might not actually work, you might end up in theatre, that’s never said to them, it’s just…the doctor is doing them a favour, ‘we’re going to induce you, it’s going to be great,’ you know. And they’re never told that it could actually all go pear-shaped and you could end up in theatre afterwards. (Clinicians FG 1)*

Enhanced communication was particularly relevant to women who were considering a VBAC. They readily acknowledged that open communication with clinicians enhanced their confidence in VBAC, and clinicians also recognised the importance of communicating with women immediately post the first CS:

*One of the girls in my antenatal class…had the meeting, like, with the midwife, who talks through deliveries…that didn’t go according to plan. And she said that’s really beneficial…she had an emergency section and was, like, just talking through that. And she found that very positive, going into her next pregnancy. (Woman FGI 3)*

*I think they need to be targeted as soon as they’re had their, their baby by Caesarean Section. They need to be really well debriefed and they need to have time to reflect with somebody on that experience and the advice then starts then as to how they might be able to look towards having a VBAC in the future. Like, waiting for them to come back pregnant again, it’s way too late. (Clinician FGI 3)*

## Discussion

Both women and clinicians have expressed beliefs that CS has become ‘normalised’ and, as such, is contributing to rising CS rates in Ireland and is undermining confidence in normal birth. Such a perception is also leading to a lack of understanding around the risks associated with CS. In Sweden, which has low CS rates, it has been acknowledged that a belief in normal birth, and the provision of mainly midwife-led care positively contributes to reducing the rates [8]. A systematic review of factors influencing decision-making for caesarean section has also previously highlighted that clinicians were influenced in their decision-making for CS by their personal beliefs, fear of litigation, and convenience [9]. Findings from our study also reflect this, with fear of litigation and a loss of confidence in normal birth influencing clinicians’ views around CS rates.

The participants in this study believed that popular and social media showed birth by CS as easier than vaginal birth, and without many risks, a view also supported in the world literature. For example, a review of 118 articles on CS in Brazilian women’s magazines found that most did not use high quality scientific information. The depiction of CS and its effects on women was described as ‘incomplete’, and the authors believed that the articles might give women an underestimated view of the associated risks [10]. The same authors repeated the study in Spain, on 1223 articles, with similar results, showing that less than 5% of the papers reported placenta praevia in the next pregnancy, infection or haemorrhage as side effects of CS [11]. A similar review explored the depiction of CS in 81 articles, 10 videos, six birth shows, two informational leaflets and one scientific paper across three countries whose CS rates were high (56%, in Cyprus), medium (36%, in Italy) and low (16%, in Iceland) [12]. The authors found that in Iceland, the media focus was on midwife-led care and normal birth, whereas in Cyprus and Italy, the media focussed more on the need to reduce the high rates of CS. The authors suggested, based on the different types of messages in the media in the three countries, that high CS rates did not exist as a result of clinical need, but were a social phenomenon, and that the media had a significant influence on the choices made by women, their families and clinicians caring for them [12]. In addition, a study in Norway analysed newspaper coverage over a ten-year period and compared it with mode of birth for 620,000 women. The authors found that adverse publicity increased the probability of having a CS, which they believed was due to obstetricians being sensitive about having their reputations damaged by the public press [13]. These sentiments correspond with the views expressed by the clinicians and women in the present study.

Our participants (both clinicians and women) identified enhanced antenatal education as an opportunity to promote confidence in normal birth and decrease CS rates, and women expressed a desire for better preparation to increase their confidence. This accords with the most recent Cochrane review on non-clinical interventions for reducing unnecessary CS, which found evidence that childbirth training workshops for mothers alone, or couples, may reduce caesarean section and increase spontaneous vaginal birth [14].

Participants also described a need for increased resources, with a lack of staff and facilities hampering normal birth and leading to a ‘conveyer-belt’ type of care. This concurs with Panda et al’s systematic review of 34 studies from 20 countries, which reported that a lack of resources (staff, birthing rooms/beds) resulted in clinicians making the decision to conduct CSs more frequently [9]. An Australian study of the costs of a ‘Complementary Therapies for Labour and Birth’ programme of education for pregnant women found a cost saving of $A659 per woman, due to significantly fewer women in the study group having a CS [15]. Thus, any associated increased cost in improving the quality and availability (frequency, duration) of antenatal education could be covered by savings from a decreased cost of care, as is recommended by FIGO’s recent position paper on reducing CS rates [6].

Studies in England [16], Ireland [17, 18] and Sweden [8] have shown that a shared philosophy among all clinicians and women that prioritises normal birth is a key factor in reducing CS rates and maintaining them at an acceptable level. Participants in the present study echoed this belief.

Clinicians in this study believed increases in rates of IOL were contributing to increases in CS rates and that a lack of preparation among women undergoing compounded this. Women in this study did however demonstrate awareness of the potential impact of IOL on mode of birth. The literature on the effect of IOL on CS rates is mixed. The recent Cochrane review [19] shows that there is a clear reduction in perinatal death and a decrease in CS rates with IOL compared to expectant care, and a review of 101 systematic reviews of interventions to reduce CS also found that IOL decreased CS rates [19]. However, there is some work showing that, when labour is induced in nulliparous women in Australia, the CS rate increases from 12% to 22% [20] or, in Ireland, from 19% to 29% [21]. Another Irish study comparing IOL and CS rates in ‘private versus public patients’ showed that the CS rate for all women following spontaneous onset of labour was 9.22%, compared with a CS rate of 31.25% following IOL [22], which lends credence to our participants’ views. This may be a uniquely Irish problem, perhaps due to different methods used for IOL, or differing gestational ages used as a criterion for induction, as IOL with an unripe cervix is more likely to lead to a ‘failed’ induction, a principal cause of CS [23].

Women and clinicians identified practical measures to reduce CS including remaining at home in early labour and emphasis on supporting the physiological birth through effective communication and a shared philosophy among women and clinicians. Poor communication has been noted in many studies around the world [24–26] despite the fact that good communication has been identified as one of the key elements of respectful care in childbirth [27]. Continuous support in labour, which usually involves a good, communicative relationship (whether with healthcare provider or partner), leads to an increase in spontaneous birth [28]. Shared decision-making has been identified as vitally important in pregnancy and throughout labour and birth, with an emphasis on equality in discourse enabled by the clinician [29]. The fact that clinicians in this study did not express the need for enhanced communication or shared decision-making with women may indicate a lack of understanding of what key improvements are needed in their practice.

This study provides valuable insights from women on clinicians on what they consider will reduce CS rates, nevertheless some limitations also must be acknowledged. The setting for this study in a single large tertiary maternity hospital may limit transferability of the findings to other settings. However we do note that our findings have previously been noted in other settings; such as belief that a lack of resources and shared philosophy between women and clinicians contributes to increased CS rates [8, 9]. Another potential limitation of this study is the setting for the conduct of the clinician FGIs. Conducting these on-site may have limited the ability of many clinicians in particular to participate. This limitation was also apparent during the FGIs when clinicians were sometimes called away to answer bleeps or had to leave the interview early. However, the findings remain important and reinforce the lack of resources which they reported in the FGIs. Future studies will need to consider data collection strategies which maximise participation while supporting clinicians to attend.

## Conclusion

The belief that CS has become ‘normalised’ is prevalent, and popular and social media support and exacerbate this view. A shared philosophy that prioritises normal birth is a key factor in reducing CS rates; however, clinicians express fears around litigation and its effect on their reputation that may lead to defensive practice.

Providing the optimum birth environment for women to achieve normal birth can be challenging in the current context in which care is offered and many of the changes suggested by clinicians will require considerable support, investment and strong leadership. Changing the culture that has ‘normalised’ CS, by listening to the shared views of clinicians and women, is a critical step towards reducing unnecessary caesareans safely and supporting normal birth. Pregnant women and clinicians have offered practical solutions such as: decreasing induction of labour rates; enhancing antenatal education; offering strategies to support normal birth to all women; improving communication between clinicians and women; and increasing shared decision-making. Many of these recommendations have already been shown to reduce rates of intervention and enhance normal birth rates and could be funded by savings from the decreasing CS rate. There is a wealth of research evidence available demonstrating how CS rates may be reduced; it is now time for global and regional action to reverse the unprecedented rise in CS rates.
